# Assessment of dynamic change in psychotherapy with asdolescents

**DOI:** 10.1186/s13034-018-0246-z

**Published:** 2018-07-30

**Authors:** Elisabeth Ness, Hanne-Sofie Johnsen Dahl, Peter Tallberg, Svein Amlo, Per Høglend, Agneta Thorén, Jens Egeland, Randi Ulberg

**Affiliations:** 1 Division of Mental Health and Addiction, University of Oslo, Oslo, Norway; 2 Research Unit, Division of Mental Health, Vestfold Hospital Trust, PO Box 2168, 3103 Tønsberg, Norway; 3 Research Unit, Division of Mental Health, Østfold Hospital Trust, PO box 300, 1714 Grålum, Norway; 4 Dragonveien 24, 1396 Billingstad, Norway; 5 The Erica Foundation, Stockholm, Sweden; 6 Department of Psychology, University of Oslo, Oslo, Norway

**Keywords:** Rating scales, Outcome, Adolescent, Short-term psychodynamic psychotherapy (STTP)

## Abstract

**Background:**

Diagnostic interviews and questionnaires are commonly used in the assessment of adolescents referred to child and adolescent mental health services. Many of these rating scales are constructed for adults and focus on symptoms related to diagnosis. Psychodynamic Functioning Scales (PFS) focus on relational aspects and how the patients handle affects and solve problems, rather than manifest symptoms. As these aspects are considered important for mental health, the PFS were developed to assess change in adults, consistent with the relational and intrapsychic concepts of dynamic psychotherapy. The scales describe internal predispositions and psychological resources that can be mobilized to achieve adaptive functioning and life satisfaction. PFS consist of six subscales; the relational subscales *Family*, *Friends* and *Romantic/Sexual relationships* and the dynamic subscales *Tolerance for Affects*, *Insight* and *Problem*-*solving Capacity*. PFS has been used for the first time as a measure of change in adolescent psychotherapy. This study examines the reliability of PFS when used to assess adolescents’ level of relational functioning, affective tolerance, insight, and problem-solving capacities.

**Methods:**

Outpatient adolescents 16–18 years old with a major depressive disorder were included in the First Experimental Study of Transference work in Teenagers (FEST-IT). They were evaluated before and after time-limited psychodynamic psychotherapy with an audio-recorded semi-structured psychodynamic interview. Based on the audio-tapes, raters with different clinical background rated all the available interviews at pre-treatment (n = 66) and post-treatment (n = 30) using PFS. Interrater reliability, the reliability of change ratings and the discriminability from general symptoms were calculated in SPSS.

**Results:**

The interrater reliability was on average good on the relational subscales and fair to good on the dynamic subscales. All pre-post changes were significant, and the analyses indicated discriminability from general symptoms. The interrater reliability on PFS (mean) and Global Assessment of Functioning were good to excellent.

**Conclusion:**

Based on the interrater reliability in our study, PFS could be recommended in psychotherapy with adolescents by experienced clinicians without extensive training. From the post-treatment evaluations available, the scales seem to capture statistically and clinically significant changes. However, the interrater reliability on dynamic subscales indicates that subscales of PFS might be considered revised or adjusted for adolescents.

*Trial registration* First Experimental Study of Transference-Work-In Teenagers (2011/1424 FEST-IT). ClinicalTrials.gov Identifier: NCT01531101

**Electronic supplementary material:**

The online version of this article (10.1186/s13034-018-0246-z) contains supplementary material, which is available to authorized users.

## Background

Assessing psychological growth in adolescents is important to identify whether a specific treatment is effective. The average treatment effectiveness is important, although to individualize treatment and help those who don’t improve, research should also focus on the mechanisms underlying treatment effectiveness [1].

Therapy with adolescents presents the therapist with specific challenges that might be different from psychotherapy with adults. Establishing a therapeutic alliance with adolescents could be comprised by the patients seeing the therapist as just another authority figure in their lives [2]. Adolescents are at a stage in their development in which they are struggling with autonomy and individuation, and they need to undertake several developmental tasks to make a successful transition to adulthood. The ability to recognize and process emotions is under development [3, 4]. In addition, dropout from treatment is significant, especially for adolescents [5, 6].

There is emerging evidence of the efficacy of psychodynamic psychotherapy for children and adolescents [7, 8]. A recent randomized controlled superiority trial in England (IMPACT-study) for adolescents with unipolar major depressive disorder compared Cognitive Behavioural Therapy (CBT) and short-term psychoanalytical psychotherapy (STPP) versus a brief psychosocial intervention [7]. They concluded that none were superior to the others.

Psychodynamic psychotherapy focuses on relational and internal psychological growth. The dynamic processes one seeks to enhance during therapy includes emotional growth, development, and maturation. The normal development in young people, like growth in size, sexual maturity, emotional development, and cognitive capacity, may be potential triggers or amplifiers of psychiatric disorder, or a potential for the adolescents’ subjective quality of life.

Psychodynamic therapy aims at helping patients understand more of the origin of their symptoms, and the function these symptoms may play in their life. In addition, self-understanding of interpersonal patterns is seen as a central change mechanism in dynamic psychotherapy [9, 10]. The achievement of a more nuanced understanding of self and others might enhance psychological flexibility without developing symptoms.

Clinicians and researchers are interested in the therapeutic effect on recurrence risk and the long-term effectiveness of existing treatments. Patients who receive psychodynamic therapy seem on average to maintain therapeutic gains and appear to continue to improve after treatment ends [11, 12]. Since psychodynamic therapy aims at endowing patients with healthier relationships, greater insight and increased awareness of their affects, psychodynamic therapy may contribute to the prevention of recurrent symptoms also in therapy with adolescents.

Diagnostic interviews and questionnaires are commonly used in the assessment of young people referred to child and adolescent mental health services. They are mainly concerned with measuring symptoms to establish diagnoses. Many psychiatric rating scales were originally constructed for adult patients and have not been tested for reliability or validity in adolescents. Over 100 different measures for evaluation of outcomes exist (reflecting upon progress in therapy, overall outcome or specific symptoms) [13]. As for self-reports, a review of child self-report measures in child and adolescent mental health services (CAMHS) identified 11 measures having potential for use as outcome measures in routine practice. However, none of these measures had sufficient psychometric evidence available to demonstrate that they could reliably measure both severity and change over time [14].

In a review of the evidence base of psychodynamic psychotherapy for children and adolescents [8], several outcome measures were used in multiple studies. There are however limitations in the existing global impairment measures. Most are unidimensional and many incorporate symptomatology into the measurement, mixing severity of psychopathology with functional impairment. Some are lengthy and thus impractical for clinical or research use. Overall global functioning measures may not differentiate what is specific for psychodynamic psychotherapy, for instance the quality of relations to close others, and the ability to think about and handle problems, as well as toleration of affects.

Fine-graded scales are needed to measure change in psychotherapy. The scales need to capture the status prior to treatment, ideally also track the improvement during therapy, and after the psychotherapy. Psychodynamic psychotherapy aims a gaining insight into the patients’ life histories and their present-day problems and to recognize non-healthy recurring patterns. The symptoms themselves are not the main focus when assessing change and outcome in dynamic psychotherapy. Although outcome measures related to dynamic capacities already exist, they tend to include a defined capacity (e.g. Reflective Functioning Scale [15]), or capacities as one aspect of comprehensive diagnostic systems (e.g. Mental Functioning Scale of the Psychodynamic Diagnostic Manual (PDM [16]), the Operationalized Psychodynamic Diagnoses (OPD [17] and the Shedler-Westen Assessment Procedure with 200 items (SWAP-200) [18]). The Wallerstein’s Scales of Psychological Capacities (SPC) is an instrument developed to meet clinical and research needs in assessing change in patients who have undergone long-term psychodynamic or psychoanalytic therapy [19]. The SPC, though rather comprehensive with 17 defined capacities, have been adapted to adolescents (Ad-SPC) [20].

To our knowledge there is a lack of brief clinician-rated instruments to assess dynamic capacities with adolescents. The Youth Outcome Questionnaire (YOQ) [21] is a 64 item report for children and adolescents (ages 4–17) completed by the parent/guardian. A self-report version also exists. A comprehensive clinician-rated instrument to assess intrapsychic processes in children and adolescents is the Operationalized Psychodynamic Diagnoses in Children and Adolescents (OPD-CA-2) [17], a multiaxial diagnostic and classification system based on psychodynamic principles based on four axes (interpersonal, conflict, structure, and prerequisites for treatment). The diagnostic way of thinking does not require training, but the rating should ideally be done by certified raters.

### Psychodynamic Functioning Scales

In the present study we seek to test the reliability of an instrument which is developed to capture change after psychodynamic therapy. Høglend and colleagues developed a set of scales measuring psychological functioning, the Psychodynamic Functioning Scales (PFS) [22]. PFS are meant to discriminate from general symptoms or global functioning and capture the complexity of changes that potentially can occur during and after psychodynamic therapy. Ratings are based on a semi-structured dynamic interview. Current functioning within the last 3 months are rated. The clinician rated scales describe internal predispositions, psychological resources, capacities, or aptitudes that can be mobilized by the individual in order to achieve adaptive functioning and life satisfaction. The six scales are: *quality of family relationships*; *quality of friendships*; *romantic/sexual relationships*; *tolerance for affects*; *insight*; and *problem*-*solving capacity*. The scale format has been modelled after the Global Assessment of Functioning (GAF), with ten descriptive levels and scale points ranging from 1 to 100. Each of the six scales therefore covers the entire range of functioning, from superior (100) to extremely poor (1). The use of a well-known scale format should make the scales easier to learn. The intention was to make the scales “fine-grained” enough to capture reliable changes during psychotherapy. The content validity and Guttman scale structure have been tested with Q-sort methodology [23–25] performed by a large number of psychotherapists from Norway, Finland, and Germany [26]. PFS has been deemed as a reliable instrument to assess mental health and change after therapy in adults [22]. Using the Psychodynamic Functioning Scales as an outcome measure in a study of adults revealed that insight was the most difficult scale to rate reliably, especially at pre-treatment [22]. The Psychodynamic Functioning Scale has not until now been reliability-tested for adolescents.

### Aims

The present study tests the interrater reliability of five scales from PFS: Quality of Family Relations, Quality of Friendships, Tolerance for Affects, Insight and Problem-Solving Capacity. The reliability of change ratings, and the discriminability from global functioning (GAF; Global Assessment of Functioning [27]) and subjective distress (GSI; Global Severity Index from the Symptom Checklist-90 [28]), during brief dynamic psychotherapy with adolescents is also tested.

## Methods

### The First Experimental Study of Transference Work-In Teenagers (FEST-IT)

Data from FEST-IT are used. FEST-IT is a randomized, controlled study on psychodynamic psychotherapy for adolescents with depression [29].

### Patients

The patients were the first 70 adolescents included in FEST-IT. One patient withdrew the consent and three interviews were lost due to technical problems with the audio-recording. Hence, 66 patients were included in the analyses in the present study. There were 12 boys and 54 girls aged 16–18 years. The patients were recruited among adolescents with symptoms of depression referred either to private practice or child and adolescent outpatient mental health clinics in the South-Eastern Health Region, representing mainly urban and some rural areas. All patients were attending classes in lower or upper secondary school.

Adolescents with current unipolar major depressive disorder according to Diagnostic and Statistical Manual of Mental Disorders, Fourth Edition (DSM-IV; American Psychiatric Association, 2000) were included. Adolescents with generalized learning difficulties, pervasive developmental disorder, psychosis, or substance addiction were excluded. Comorbidity was expected to be frequent.

Axis I and II diagnosis were based on the Mini International Neuropsychiatric Interview (M.I.N.I.) and Structured Interview for DSM-IV Personality (SIDP-IV). Table 1 shows some of the pre-treatment characteristics. Axis I diagnoses beside depression were mostly social phobia, panic disorder and general anxiety. A total of 31 patients had one or more Axis II disorders—primarily depressive or avoidant personality disorders. The patient sample had, on the average, mild to moderate symptoms and dysfunctions. The mean GAF score at the initial psychodynamic evaluation (PFS) was 58.0 (SD = 6.1 range 44.2–73.2). The mean GSI score (from SCL-90) was 1.3 (SD = 0.5, range 0.5–2.7). The mean BDI score was 28.7 (SD = 9.0, range 10–58). The distribution of mean pre-treatment scores indicated that the sample of 66 patients was a group of moderately depressed adolescents, representative of typical outpatients offered dynamic psychotherapy. The range of the pre-treatment scores of the five scales of PFS covered the area of functioning from relatively severe and chronic disturbances to moderate and intermittent problems of living (range 45.6–71.0). Only one patient reported taking antidepressant medication at baseline, i.e. at the beginning of therapy. One patient was taking antidepressants at the end of therapy. This was, however, not the same patient. One patient was taking antipsychotics throughout the study period. One patient was taking sleeping medicine at pre-treatment and 4 patients were taking sleeping medicine at post-treatment.

**Table 1:** Pre-treatment characteristic of the 66 patients included

	Total (n = 66)
	Mean (SD)
Age	17.3 (0.7)
PFS	59.7 (6.1)
GAF	58.0 (6.1)
IIP-C	1.36 (0.4)
GSI	1.33 (0.5)
BDI	28.7 (9.0)

	N (%)
Female	54 (82)
Axis I diagnoses	
Depressive disorder	100 (100)
Social phobia	19 (29)
Panic disorder	13 (20)
General anxiety	17 (26)
Eating disorder	2 (3)
PTSD	2 (3)
More than two axis I diagnoses	17 (26)
Axis II diagnoses	30 (45)
Depressive	24 (36)
Avoidant	19 (29)
Negativistic	4 (6)
Obsessive compulsive	3 (5)
Paranoid	3 (5)
Dependent	2 (3)
Borderline	1 (2)
Histrionic	1 (2)
Schizoid	1 (2)
More than one axis II diagnoses	17 (26)

*PFS* Psychodynamic Functioning Scale; *GAF* Global Assessment of Functioning (n = 47), *IIP-C* inventory of interpersonal problems—circumplex version, *GSI* Global Severity Index (SCL-90), *BDI* Beck Depression Inventory

### Therapists

The twelve therapists worked in out-patient clinics and/or in private practice. Eight were psychiatrists and four were clinical psychologists. There were six men and six women. All therapists were trained therapists and had at least 2 years of formal training in psychodynamic psychotherapy.

### Treatment

Short-term psychodynamic/psychoanalytic psychotherapy (STPP) based on the STPP manual from the IMPACT study [30] was used as the manual for the treatment. The manual combines aspects of STPP that focus principally on techniques aimed at helping young people overcome developmental problems, as well as emphasizing the role of the interpretation of unconscious conflicts, attachment theory and the concepts of internal working models. With the agreement of the adolescent, parallel work with parents was included. Antidepressant medication could be added in severe cases according to the national guidelines in Norway [31]. The patients were randomized to two treatment groups. In both groups general psychodynamic techniques [30] were used. The patients were offered 28 weekly sessions.

A 1-year training program prepared the therapists for treating patients in the study. Peer supervision in groups with material from the audio-recorded therapies was offered regularly during the study to help maintain the quality of the therapies and adherence check to the manualised therapies.

### Evaluators and raters

Four individual evaluators conducted the patient interviews at baseline (pre-treatment) and at the end of therapy (post-treatment). The four evaluators and the two raters were clinical psychologists or psychiatrists and had their clinical training from different psychodynamic institutes. The four evaluators were females, while the two raters in this study were males. All had long clinical experience ranging from 12 to 30 years. One of the two raters had his main clinical background from out-patient adults, while the other rater had been working with adolescents from an in-patient department over the last decade.

Both the evaluators and raters were blind to treatment. They met on regular basis for group supervision both before and during the study. Meetings also involved plenary discussions after individual scorings of audio-recorded interviews.

### Measures in the present study

#### Psychodynamic Functioning Scales (PFS)

PFS [22] were developed to capture evaluator-rated change in dynamic and interpersonal functioning. Current functioning was rated on the basis of a semi-structured dynamic interview. Five of the six scales were used: Quality of Family Relations, Quality of Friendships, Tolerance for Affects, Insight and Problem-Solving Capacity. The five subscales used in the analysis are presented in the Additional file 1. Each of the scales covers the entire range of functioning, with ten descriptive levels and scale points ranging from 1 to 100. The relational scales, *quality of family relations* and *quality of friendships* and *romantic/sexual relationships*, cover the mutuality and emotional responsiveness in relationships. The ratings of the two scales related to family and friends are based on evaluating the degree of mutuality and adequacy of the commitment in relationships, the ability to take other’s perspective, to describe close others across an external and internal dimension, feeling of being needed and a sense of belonging and the capacity to reconcile parent’s or friends’ shortcomings and make the best of the relationship. If parents are not alive the evaluation is based on memory of them or internalized object relations. The *romantic/sexual relationships* involve also the capacity to establish long-term relationships characterized by love, trust, reciprocal mature dependency and active, flexible sexual pleasure. The *tolerance for affects* covers the ability to experience, differentiate and express various affects verbally and nonverbally, and to what degree disappointments lead to symptoms like avoidance, anxiety, depression or restrictions of goals. *Insight* covers mainly cognitive understanding of the main dynamics of inner conflicts, related inter-personal patterns and connection to the past. Also, the ability to describe and understand own vulnerability and reactions to stress. The *problem*-*solving capacity* covers the ability to handle any difficult situation without developing symptoms, avoidance or inadequate actions. Self-observation, planning, ability to explore new areas and enjoy recreation and pursue meaningful goals are parts of this scale. The PFS is deemed to be reliable [22, 26]. Although most adolescents have some experience of intimate relations, the minority have yet established more definite intimate relationships patterns. Thus, the scale *romantic/sexual relationships* was omitted for adolescents in the present study. The scales (Additional file 1) were developed with descriptive levels in English. In FEST-IT the English version was used although the semi-structured interview with anchor points was in Norwegian.

#### Global Assessment of Functioning (GAF)

The GAF (DSM.3rd ed. 1987) [27] is a numeric scale (1 through 100) with ten descriptive levels assigning a clinical judgment to the individual’s overall functioning level. GAF recorded values used in FEST-IT are separate scores for symptoms (GAF-S) and functioning (GAF-F). For both the GAF-S and GAF-F scales, there are 100 scoring possibilities (1-100). Impairments in psychological, social and occupational/school functioning are considered, but those related to physical or environmental limitations are not. GAF seek to capture symptom relief. GAF was an outcome measure in the adult study FEST and therefore chosen also in the adolescent study instead of Children’s Global Assessment Scale (CGAS). The GAF-scale can be scored reliably although the limitations as a single instrument has been discussed. [32, 33].

#### Symptom Checklist-90 (SCL-90)

The SCL-90 [28] is a self-report psychometric instrument (questionnaire) designed to evaluate a broad range of psychological problems and symptoms of psychopathology. It is also used in measuring the progress and outcome of psychiatric and psychological treatments or for research purposes. The SCL-90-R is normed on individuals 13 years and older. It consists of 90 items and takes 12–15 min to administer. The SCL-90 is used as an outcome measure in many studies. In the present study we use the General Symptom Index, which is the mean of the 90 items. Its psychometric properties have been examined and described [34, 35].

#### Beck depression inventory (BDI-II)

The BDI-II [36] is a widely used 21-item self-report inventory composed of items relating to symptoms of depression. The BDI-II is designed for individuals aged 13 and over, thus measuring the severity of depression in adolescents and adults. Psychometric properties have been described with high reliability and a capacity to discriminate between depressed and non-depressed subjects and high content and structural validity [37].

#### Evaluation and rating

Each patient was interviewed by one evaluator at pre- and post-treatment with a semi-structured GAF interview and a psychodynamic interview modified after Malan [38] and Sifneos [39]. The psychodynamic interview lasted approximately 45–60 min and the therapist was present if possible. However, the rater did not discuss or clarify questions with the therapist during the interviews or before rating the scales. No therapist ratings were included in the analysis. Ratings on the five dynamic scales and GAF were done by the evaluator. After the interviews, the patients filled out the SCL-90-R and the BDI-II. All interviews were audio-recorded and independently assessed by two additional raters. During plenary calibration meetings after the individual ratings were recorded, the ratings and quality of the interview was discussed.

#### Statistical analysis

The raters and evaluators assessed the patients before and after therapy. From this group of six we estimated the interrater reliability (IRR) for single raters at pre-treatment. Assessments by the two raters were used to determine the IRR at pre- and post-treatment. Ratings of audio-recorded interviews rated by the same two raters for all subjects (66 at pre-treatment and 30 at post-treatment) were used for the Intra Class Correlation-analyses (ICC) [40] (two-way mixed consistency) for ordinal scores. This is represented in SPSS as “Two-Way Mixed” because it models both an effect of rater and of ratee (i.e. two effects) and assumes a random effect of ratee but a fixed effect of rater (i.e. a mixed effect model). The statistical analyses were done using SPSS version 23 SPSS.Inc. 2016. Ratings of GAF were only available for analysis in 47 patients pre-treatment due to missing data. Only 30 patients were rated on both occasions by both raters. The pre-/post-ratings include the same 30 patients for all instruments including GAF.

We also estimated the ICC for average scores of 3 raters, including the evaluator for each subject as the third rater, at pre-treatment. The model was then “Two-Way Random” in SPSS.

Average pre-treatment scores on each scale were compared with average post-treatment scores, by use of paired t-tests, on the 30 patients evaluated before and after therapy from 3 raters.

Guidelines for evaluating assessment instruments in psychology developed by Cicchetti and Sparrow [41], closely resembled by guidelines by Fleiss [42] and by Landis and Koch [43], state that when the reliability coefficient is below 0.40, the level of clinical significance is poor; when it is between 0.40 and 0.59, it is fair; when it is between 0.60 and 0.74, it is good; and when it is above 0.75 the level of clinical significance is excellent.

Jacobson and Truax [44] have developed a commonly used measure of assessing statistically reliable change-the Reliable Change Index (RCI). The RC coefficient is equivalent to the difference between two scores divided by the standard error of the difference between the scores, which is derived from test–retest reliability of a measure and standards deviation of pre-treatment scores on that measure (RCI = (X_post − _X_pre_)/S_diff_) where S_diff_ = the standard error of the difference between the two test scores. S_diff_ = √S(SE_m_^2^) and the Standard Error of the Measurement SE_m_ = s√1 − r_xx_ where r_xx_ = reliability coefficient of the instrument (in this study the ICC was used). For the GAF, GSI and BDI the S_diff_ were calculated from applying the denominator from the *t* test formula with *s*_1_ and *s*_2_ as variance of the pretest scores and posttest scores. An RC coefficient that is larger than 1.96 is usually regarded as unlikely (p < 0.05) to occur without any actual change and an indication of the individual’s reliable change.

We used a SPSS correlation test (Pearson correlation) to estimate if the average scores of pre-treatment variables were discriminable from general symptoms (GSI) or dysfunction (GAF).

## Results

The interrater reliability estimates of all the patients available for analysis at pre-treatment for single raters are shown in Table 2.

**Table 2:** Interrater reliability estimates (intraclass correlation; ICC) for single raters randomly drawn from a group of six (4 evaluators and 2 raters)

PFS pre-treatment (n = 66)	ICC	95% CI
Family	0.60	0.46–0.71
Friendships	0.56	0.43–0.69
Tolerance for affect	0.44	0.29–0.58
Insight	0.46	0.31–0.60
Problem-solving capacity	0.56	0.42–0.69
PFS mean	0.61	0.47–0.72
GAF pre-treatment (n = 47)	0.66	0.52–0.78

*PFS* Psychodynamic Functioning Scales, *GAF* Global Assessment of Functioning

To study reliability in subscales we report the ICC for each subscale of PFS, mean values of PFS, and GAF. The lower bounds of the confidence intervals were unsatisfactory (< 0.40) for the subscales *tolerance for affects* and *insight*. With the average scores of the two raters who rated all subjects, the interrater reliability estimates of the 66 patients pre-treatment and of 30 of the same patients from post-treatment are shown in Table 3. At pre-treatment, we achieved excellent average reliability on the scales *family, friendships*, *PFS mean* and *GAF*, and good reliability on *insight, tolerance for affects* and *problem*-*solving capacity*. The lower bound of the confidence interval was unsatisfactory for only one of the single scales at pre-treatment: *insight*.

**Table 3:** Pre-treatment and post-treatment interrater reliability estimates (intraclass correlations; ICC), for average scores of two raters

PFS	Pre-treatment (n = 66) ICC		Post-treatment (n = 30) ICC	
Family	0.82**		0.66	
Friendships	0.75*		0.73*	
Tolerance for affect	0.69*		0.80*	
Insight	0.61		0.59	
Problem-solving capacity	0.70*		0.71*	
PFS mean	0.81**		0.80*	
GAF	0.80**^a^		0.87**	

* Lower bounds of 95% confidence interval (CI) > 0.40 ** lower bounds of 95% CI > 0.60

^a^ Pre-treatment n = 47

At post-treatment, also Table 3, with the average scores of the two raters, the ICC measures were all above 0.60 except for the subscale *insight* (0.59). However, the lower bounds of the confidence intervals were less than 0.40 for 2 subscales: *family* and *insight*.

The two raters based their ratings on audio-recorded interviews only. The evaluators, on the contrary, met the patients for interviews as part of the assessment. At pre-treatment, the two raters differed in rating the relational and dynamic subscales respectively. The one rater tended to rate the patients higher than the other rater on the relational scales; *family* and *friendships*. However, the situation was quite opposite for the dynamic scales; *tolerance for affects*, *insight* and *problem*-*solving capacity*. On all the dynamic subscales, the other rater was rating the patients with higher scores.

With ratings also from the evaluators interviewing the patients at pre-treatment, the interrater reliability estimates increased. For all subscales of PFS, lower bounds of the 95% confidence intervals were above 0.50 and all ICC-values ≥ 0.70. The same was true for GAF (Table 4).

**Table 4:** Pre-treatment interrater reliability estimates (intraclass correlations; ICC), for average scores of 3 raters (evaluator and 2 raters)

PFS pre-treatment (n = 66)	ICC	95% CI
Family	0.82	0.72–0.88
Friendships	0.79	0.69–0.87
Tolerance for affect	0.70	0.55–0.81
Insight	0.72	0.58–0.82
Problem-solving capacity	0.79	0.69–0.87
PFS mean	0.82	0.73–0.89
GAF pre-treatment (n = 47)	0.85	0.76–0.91

*PFS* Psychodynamic Functioning Scales, *GAF* Global Assessment of Functioning

Table 5 presents the mean scores on all subscales of PFS at pre-treatment and post-treatment for the 30 patients evaluated on both occasions. The post-treatment values of PFS and GAF indicate less severe problems in psychodynamic functioning and global symptoms at the end of therapy. The decreased post-treatment values of BDI and GSI indicate less depressive symptoms and less symptoms of psychopathology respectively. All changes were statistically significant at p < 0.01 paired t-test (two-tailed). The largest amount of change on the PFS subscales during the individual psychotherapy, and the highest ratio of patients with reliable changes according to the Reliable Change Index (RCI) [44], tended to be for the *tolerance for affects* (10 patients). The RCI was equal for PFS mean and GAF. The cut off score for reliable change was 6.1 for PFS mean and 6.8 for GAF, meaning a patient would have to improve with more than 6.1 points on the mean rating of PFS for the change to be considered reliable. The individual improvement seemed to be most reliable for the self-reported depression scale BDI (74% of all cases analysed).

**Table 5:** Changes from pre-treatment to post-treatment with average scores of 3 raters (evaluator and 2 raters), (n = 30)

Scale	Mean ± SD		Sig. (2-tailed)	Reliable change index (% of all cases)
	Pre-treatment	Post-treatment		
Family	63.3 ± 7.1	66.3 ± 7.1	< 0.001	17
Friendships	66.1 ± 7.6	70.2 ± 6.5	0.008	17
Tolerance for affect	56.8 ± 4.7	62.8 ± 5.7	< 0 .001	33
Insight	59.3 ± 5.9	65.3 ± 5.8	< 0.001	30
Problem-solving capacity	59.8 ± 5.1	64.7 ± 5.9	< 0 .001	30
PFS mean	60.8 ± 5.2	66.0 ± 5.3	< 0.001	40
GAF	59.2 ± 5.5	67.1 ± 7.4	< 0.001	40
BDI	29.2 ± 7.3	15.1 ± 12.5	< 0.001	74
GSI	1.31 ± 0.5	0.81 ± 0.5	< 0.001	60

*PFS* Psychodynamic Functioning Scales, *GAF* Global Assessment of Functioning, *BDI* Beck Depression Inventory, *GSI* Global Severity Index

A correlation matrix was made to evaluate whether the subscales from PFS could be differentiated from global functioning and subjective distress. Table 6 shows the results. *Tolerance for affects* and *problem*-*solving capacity* seemed to be the subscales with the highest correlation with GAF. The correlation with GSI was weak or moderate for all subscales. The PFS mean had a strong correlation (0.72) with GAF and a weak correlation (− 0.29) with GSI.

**Table 6:** Pearson’s correlations of PFS subscales (n = 66), GAF and GSI

Variable	1	2	3	4	5	6	7	8	
1. Family	–								
2. Friendships	0.52**	–							
3. Tolerance for affects	0.55**	0.65**	–						
4. Insight	0.42**	0.47**	0.77**	–					
5. Problem-solving capacity	0.46**	0.62**	0.81**	0.73**	–				
6. PFS mean	0.75**	0.83**	0.90**	0.80**	0.85**	–			
7. GAF (n = 47)	0.48**	0.48**	0.74**	0.63**	0.73**	0.72**	–		
8. GSI (n = 57)	− 0.33*	− 0.08	− 0.32*	− 0.04	− 0.41**	− 0.29*	0.39**	–	

*PFS* Psychodynamic Functioning Scales, *GAF* Global Assessment of Functioning, *GSI* Global Severity Index

* Correlation is significant at the 0.05 level (2-tailed)

** Correlation is significant at the 0.01 level (2-tailed)

## Discussion

The interrater reliability of the PFS for assessment of change in psychodynamic therapy with adolescents was on average good on the subscales *family* and *friends* (relational subscales), and fair to good on the subscales *tolerance for affects*, *insight* and *problem*-*solving capacity* (dynamic subscales).

The two raters at pre-treatment differed in rating the relational subscales and the dynamic subscales. They tended to rate respectively relational and dynamic scales higher or lower than the other rater. The difference was not so clear at post-treatment, although the interrater reliability did not change. The interviews regularly revealed whether the adolescents had been in therapy or not. The raters were therefore not totally blind regarding whether the evaluation was pre-treatment or post-treatment. However, the two raters were scoring pre-and post-treatment interviews randomly and the chronology of ratings is not parallel to the therapies.

With the average scores of three raters (evaluator and 2 raters), the IRR was good to excellent for all subscales. Høglend and colleagues in the adult FEST-study achieved good results for all single scales at pre-treatment as well as post-treatment with average scores of three raters [22]. In the present study, *insight* was, as in the adult study, the most difficult scale to rate reliably. In relation to psychoanalytic theory, dynamic insight is a measure related to subjective interpretation and understanding of symptoms, vulnerabilities and strengths. Adolescents progress at varying rates in developing their abstract and reflective thinking ability. They may also be more reluctant to talk about the past and link today’s problems with past experiences. Only fair to good reliability was achieved also on the scale *problem*-*solving capacity*. Some adolescents may be able to apply logical operations long before they are able to apply them to personal dilemmas. When emotional issues arise, they often interfere with the young’s ability to think in more complex ways. The ability to consider possibilities, as well as facts, may influence decision-making, in either positive or negative ways. This might result in incoherent information and descriptions from the adolescents themselves.

The ratings of subscales were based on the original English version of PFS. Although the dynamic interviews with anchor points relating to each subscale were conducted in Norwegian, i.e. the mother tongue of the evaluators and raters, the use of scales in English might influence the ratings and the interrater reliability.

The dynamic scales seemed harder to score in agreement than the relational scales. However, the changes from pre- to post-treatment were larger for the dynamic scales. An explanation might be that more nuanced post-treatment information contributed to relatively lower ratings of relationships to family and friends. However, the larger change on dynamic scales would be consistent with the concepts of dynamic psychotherapy. It might be explained by the possibility that the patients had been better acquainted with the concepts of therapy and that the therapy mirrored new ways of understanding and talking about perceived problems. The time-period during therapy is though important time for development. From the post-treatment evaluations available, the scales seem to be sufficiently fine-graded to capture statistically and clinically significant pre-post changes during time-limited psychodynamic psychotherapy with depressed adolescents. However, because of small sample size the results may be unstable.

The dynamic scales seem to measure a construct that may prove discriminable from general symptoms and dysfunctions. The PFS mean value and GAF correlated strongly. The correlation with GSI (global symptoms) was weak.

The reliability change estimates for individual scales in our study is similar to the adult study. This is however true for the PFS. The RCI was larger for the self-reported symptom scores (GSI) in our study. The mean GSI was higher at pre-treatment for adolescents (1.3 vs 1.0). It may indicate that adolescents on self-reports tend to be more extreme in reporting symptoms compared to adults. The evidence base for psychodynamic psychotherapy for children and adolescents is building up [45], but e.g. there are few instruments for assessment of intrapsychic processes specific to the treatment. Outcome evaluation forms may add information to the therapists about their effectiveness and also be of value to researchers examining underlying mechanisms to explain outcome [46].

Although the PFS is used as an outcome measure, it might be useful in further process-outcome studies regarding mechanisms and moderators through which treatment interventions operate [47]. Variations in outcome seem to be influenced by patient characteristics and by the therapist variables and context factors [48–50]. PFS has no parallel forms to compare patient’s and therapist’s ratings, often used in process research (e.g. the rather newly developed Individual Therapy Process Questionnaire (ITPQ) [51]). Observer-ratings might still be a strength. In other studies of depression, both clinician-rated and self-reported instruments are recommended, although primarily related to symptoms [52, 53].

Reliability is important for outcome assessment. Although an assumption that stable and durable changes in personality characteristics are the most difficult to detect, changes in less stable variables such as mood and affect may have larger effect sizes in response to treatment—but lower reliability estimates. Following measurements administered during and after treatment with statistically significant differences would implicate measures sensitive to change. However, if not compared to other outcome measures there might be a lack of evidence that the real change occurred. Assessment of change makes further investigation of change possible and contributes to the field of research linking to clinical utility in the ideal personalization of psychotherapy treatment for adolescents. The ratings on PFS are based on the last 3–4 months and therefore the instrument might serve best as a follow-up in therapies lasting 6 months or more.

In plenary meetings trainees and students were often present. They were seldom the “outliers” and we were surprised how relatively easy it was for them to grasp the concept of the rating scales. The interrater reliability in this study on adolescents suggests that the PFS could be used with adolescents in psychotherapy by experienced clinicians without extensive training.

Romantic and/or intimate relationships are important to adolescents and this scale should be considered revised and adjusted for adolescents to capture the ability to establish and stay in intimate mutual relationships.

### Limitations

The small sample size and the fact that data from only half of the included patients was available for post-treatment analysis are both limitations of this study. The interrater reliability of additional raters attending plenary meetings are not analysed and reported. However, preliminary results are promising. Analyses on a full dataset would improve the statistical power.

## Conclusion

Based on the interrater reliability (IRR) results in our study, the PFS could be recommended for use in psychotherapy with adolescents by experienced clinicians without extensive training. The IRR was good to excellent for all five subscales with the average scores of three raters. The scales seem to capture statistically and clinically significant changes. However, the IRR on the dynamic subscales *tolerance for affects*, *insight* and *problem*-*solving capacity* indicates that subscales of PFS might be considered revised or adjusted for adolescents.

## Additional file

**Additional file 1.** Scales developed to assess change in dynamic psychotherapy.

## Abbreviations

PFS: Psychodynamic Functioning Scales
FEST-IT: First Experimental Study of Transference Work-In Teenagers
GAF: Global Assessment of Functioning
CBT: cognitive behavioural therapy
STPP: short-term psychoanalytical psychotherapy
GSI: Global Severity Index
CAHMS: Child and Adolescent Mental Health Services
SDQ: Strengths and Difficulties Questionnaire
SIS-CA: Severity of Impairment Score for Children and Adolescents
CGAS: Children’s Global Assessment Scale
GBOM: Goal Based Outcome Measure
CBCL: Child Behaviour Checklist
BIS: Brief Impairment Scale
M.I.N.I.: Mini International Neuropsychiatric Interview
SIDP-IV: Structured Interview for DSM-IV Personality
SCL-90: Symptom Checklist-90
BDI(-II): Beck Depression Inventory
IRR: interrater reliability
ICC: Intraclass Correlation Coefficient
RCI: Reliable Change Index
CI: confidence interval
FEST: First Experimental Study of Transference Interpretations
