# Supplementary material for: Assessment of dynamic change in psychotherapy with asdolescents
Source: Child Adolesc Psychiatry Ment Health. 2018 Jul 30;12:39. doi: 10.1186/s13034-018-0246-z (PMC6065153; doi:10.1186/s13034-018-0246-z)
Supplement: Supplementary file 1 — Additional file 1. Scales developed to assess change in dynamic psychotherapy. [file 13034_2018_246_MOESM1_ESM.docx]

Additional file 1. **Scales developed to assess change in dynamic psychotherapy**

**Quality of family relations**

Quality of relations to family and important substitutes (if parents not available). Good quality involves appropriate mutuality, ability to give and take, adequate commitment, ability to trust and be trustworthy, emotional responsiveness, ability to take the other’s perspective, ability to vividly describe close others across external and internal dimensions. Involves a feeling of being needed and a sense of belonging. If close relatives are not alive, evaluate relations based on memory/internalised object relation. The capacity to reconcile parents’ shortcomings and make the best of the relationships should be considered.

100-91 Superior quality of relations to all close family members. Relationships are secure, warm, open, with respect and concern. Subject admired for ability to be emotionally responsive and understand the perspective of others. Others are described clearly and vividly as unique individuals. Transient episodes of conflict or frustration justified by circumstances and easily resolved.

90-81 Warm, open and reciprocally rewarding relationships with family. Other family members are generally seen as accepting, trustworthy and responsive. Conflicts or frustrations justified by circumstances and easily resolved.

80-71 Good stable reciprocally rewarding relationships. Problems of short duration or limited to one significant family member. Conflicts may be painful without compromising basic commitment and security. Subject shows reasonable ability to reconcile significant family members’ shortcomings.

70-61 Some family relationships experienced as problematic by the subject, but may seem normal to others. Can describe relatives as separate beings in terms of functions and also their feelings, attitudes, and values. A tendency to be mildly self-sacrificing or exploitative, overinvolved or underinvolved, mildly suspicious or gullible, dependent or counterdependent in problematic situations. Subject may be less free because of need to oppose or comply with parent introjects.

60-51 A tendency to take controlling and/or submissive roles in family. Limited experience of warmth, openness, gratification, and trust. May avoid conflict or personal pain by keeping distance, or be passive or self-sacrificing behaviour. Describes significant others such that it is difficult to visualize and recall the person being described. May be preoccupied with family members’ shortcomings or with gaining support from parental objects.

50-41 Considerable problems relating to several family members. Self-sacrificing or exploitative, markedly suspicious or gullible, very easily upset by demands or emotionally detached from significant others. Severe difficulty understanding others or taking others’ perspective. Describes others superficially, stereotypically or markedly inconsistent.

40-31 Minimal contact with family or chronic, severely maladaptive relationships patterns. No mutual gratification; exploitation, emotional detachment, severe suspicion. Other family members described globally and concretely or from a highly egocentric perspective. May have long-term severely dependent relationship to parental figures.

30-21 Strong fear of contact. Very isolated. Some contact with family only if they are tolerant with subject.

20-11 Total withdrawal from relating to family. Extreme suspicion or delusion towards family. Cannot live with family.

10-1 Disorganized mental functioning makes communication with family impossible or dangerous.

**Quality of friendships**

Quality of non-sexual interpersonal relationships involves appropriate mutuality such as ability to give and take from others, adequate commitment, ability to trust and be trustworthy, emotional responsiveness, ability to take other’s perspective, ability to vividly describe others across external and internal dimensions. Involves a feeling of being needed and a sense of belonging.

100-91 Superior quality of relations to all close friends. Relationships are secure, warm, open, with respect and concern. Several long-term relationships and also openness towards new friends. Subject admired for ability to be emotionally responsive and understand the perspective of others. Others are described clearly and vividly as unique individuals across a wide range of internal and external dimensions. Transient episodes of conflict or frustration justified by circumstances and easily resolved.

90-81 Warm, open and reciprocally rewarding relationships with friends. Other people are generally seen as accepting, trustworthy and responsive. Conflicts or frustrations justified by circumstances and easily resolved.

80-71 Good stable reciprocally rewarding relationships. Problems of short duration or limited to one significant person. Conflicts with others may be painful without comprising basic commitment and security.

70-61 Some relationships experienced as problematic by subject, but may seem normal to others. Can describe significant others as separate beings in terms of functions and also their feelings, attitudes and values. A tendency to be mildly self-sacrificing or exploitative, overinvolved or underinvolved, mildly suspicious or gullible, dependent or counterdependent in problematic situations. May be preoccupied with gaining acceptance from some others.

60-51 Have one or more long-term friendships. A tendency to take controlling or submissive roles. Limited experience of warmth, openness, gratification and trust. May avoid conflict or personal pain by keeping distance, or by passive or self-sacrificing behaviour. Describes others such that it is difficult to visualize and recall the person being described.

50-41 Mostly short-term intermittent or distant friendships. Self-sacrificing or exploitative, markedly suspicious or gullible, very easily upset by demands, or emotionally detached. Severe difficulty understanding others or taking others’ perspective. Describes others superficially, stereotypically, or markedly inconsistent.

40-31 Minimal contact with friends. No mutual gratification; exploitation, emotional detachment, severe suspicion. Others described globally and concretely or from a highly egocentric perspective. May have long-term severely dependent relationship to parental figures.

30-21 Strong fear of contact. Very isolated. Some contact with relatives or social service if they are tolerant with subject.

20-11 Total withdrawal. Extreme suspicion or delusional influence on others. Cannot live with family.

10-1 Disorganized mental functioning makes communication impossible.

**Tolerance for affects**

This dimension covers the ability to experience, differentiate and express (verbally and non-verbally) various affects. Includes ability to establish temporal gap between feelings and execution of defences or automatic behaviour. Impulse control and frustration tolerance are part of this dimension. Variables such as alexithymia and inauthenticity are partially related to this dimension.

100-91 Unusual ability to experience the richness, differentiate accurately, and express in an adequate and varied way even the strongest affects, such as anger, sadness, contempt, fear, joy, excitement, shame, anguish and sense of attachment. High tolerance for mixed feelings and ambiguity.

90-81 Even strong affects are well differentiated and flexibly expressed. Symptoms almost never develop (anxiety, depression). Some avoidance or restriction of affect occasionally occurs under heavy stress, but generally high access to emotionality.

80-71 Can experience strong affects with a reasonable ability to differentiate and express feelings. Transient symptoms or avoidance occurs, or some restriction of goals, or diminished concentration.

70-61 Severe disappointments may lead to mild symptoms, some avoidance, restricted experience and less differentiation (frustration, worrying, uncertainty, indecisive rumination, blunted joy). Inadequate expressions (outbursts, hopelessness) and restriction of ambitions and goals occur.

60-51 Disappointments relatively often lead to restriction or denial of affects, outbursts or passive complaining, or symptoms (anxiety, depression, phobias, conversion), and less differentiation of feelings. Avoidance of expression and/or restriction of goals occur.

50-41 Potential disappointments, setbacks or changes often lead to avoidance, restriction of goals and more severe and lasting symptoms. Relatively undifferentiated experience of feelings (aloof, devastated, lifeless, frantic, defective, numb) and maladaptive expression such as hopeless complaining, or frequent inadequate outbursts, or acting out occur.

40-31 Disappointments lead regularly to despair, acting out, or severe symptoms. Lack of ability to differentiate affects.

30-21 Even minor or potential disappointments lead to severe reactions of hopelessness and despair. Chaotic expression of affects. Psychotic symptoms may develop.

20-11 Cannot express any coherent feelings, very severe acting out, manic excitement. Needs some outside assistance.

10-1 Continuously disorganized psychotic mental functioning in need of constant assistance.

**Insight**

This dimension covers mainly the cognitive understanding of the main dynamics of inner conflicts, the related interpersonal patterns and repetitive behaviours, and connection to past experiences. Ability to understand and describe own vulnerability, reactions to stress, and coping abilities.

100-91 Unusual ability to describe genuinely personal wishes, fears, defences and the related behaviour and connections to earlier (childhood) experiences. High awareness of own vulnerability, attitudes, and interpersonal patterns, secondary gains. Open and curious about and reflects on the multiple levels and meanings of experience. Realistic judgement of self and others.

90-81 Can account for inner conflicts, the related problems and repetitive behaviours and connections to earlier experience. Aware of own vulnerability and reactions to stress. A tolerant and realistic sense of self and others in interpersonal disputes. May feel disillusionment, but no bitterness or hopelessness.

80-71 Can account for most important inner conflicts, related problems and repetitive behaviour patterns, and personal attitudes. Connections to earlier experience may partly be forgotten. Aware of own vulnerability, stress reactions and coping abilities. May blame self or others too much in interpersonal disputes, but reflects freely and observe own reactions and learn from it (integration). Generally curious and tolerant. Realistic expectations about the future.

70-61 Recognizes but cannot clearly describe the complex association between past experience, inner conflicts and present problems and repetitive patterns. Reasonably aware of own vulnerability and strength and reactions to stress. Tendency to blame self or others too much in disputes. Occasionally behaviour and attitude may be unrecognized, but reflects and observes self in other areas.

60-51 Understanding of inner conflicts and associations to past and present experience and behaviour is somewhat unclear, or less emotionally integrated, or “learned”. Inadequate judgement of self and others, but ability to observe and reflect with time. Vulnerability and stress-reactions sometimes a surprise. Some defensive, unrecognized attitudes and behaviours. Rigid views of right and wrongs. May look for superficial solutions. Recognizes symptoms as sign of disturbance.

50-41 Superficial “learned” or misleading ideas of inner conflicts and past and present experience. Distortions of judgement of self versus others also when no disputes. Painful feelings accompanied by harsh self-blame or incorrectly ascribed to external factors. Little or no reflection on personal motives, unaware of important aspects of attitudes and behaviours (fundamentalism). May deny symptoms as sign of disturbance. Excessive pessimism or optimism.

40-31 Does not recognize associations between behaviour and internal dynamic components. Severely distorted perceptions/judgement of self or others. Disavows painful personal reactions. Can describe internal experiences but in stereotyped, confusing or misleading way. Denies signs of mental disturbance.

30-21 Great difficulty describing internal experiences. Do not acknowledge associations between internal experiences and own behaviour. Severe distortion/delusional ideas may be present.

20-11 Disorganized or fragmented mental functioning. Breakdown of reality testing. Need outside assistance.

10-1 Continuously disorganized in need of constant assistant for days.

**Problem solving and adaptive capacity**

This dimension covers the ability to flexibly handle any difficult situation and assert self without developing symptoms, avoidance or inadequate actions. Self-observation and planning may or may not be used to enhance performance. Ability to integrate the habitual and also explore new areas, indulge with pleasure in playful activities and recreation, and pursue meaningful goals.

100-91 Unusual ability for resourceful and flexible problem solving in all areas, career, current family, family of origin, friends, leisure time, life goals. Admired for warmth, integrity, wisdom, initiative and joyfulness.

90-81 High adaptive functioning in all areas. May sometimes feel apprehensive or discouraged in difficult situations, but uses self-observations, reflection, affiliation and planning to solve problems; humour, creativity.

80-71 May occasionally feel anxious or tend to avoid critical situations. May back off or be overcompetitive but worried in situations of rivalry. Sometimes curbs own ambitions or is driven towards overachievement. Sense of direction and pursuit of goals sometimes unclear. May stay too much with the habitual or tries to explore too many new areas. Engages with pleasure in social and recreational activities.

70-61 Sometimes anxious or depressed in critical situations. Occasional inadequate actions in response to stress (aggressive or inhibit appropriate anger). May avoid one or two areas such as fails to apply for promotions or unable to change an unsatisfactory intimate relationship. May have few hobbies or interests or somewhat inhibited pleasure in recreational activities. May confine activities to the habitual and well known, or indulge in too many new areas.

60-51 Develop symptoms, avoids or acts inappropriately (aggressively or submissively) in critical and difficult situations or fails to pursue meaningful goals. Do not dare to initiate desired romantic relationships or fails to pursue realistic career goals. May show rigidity or continue maladaptive habits or fails to free self from inhibiting, or destructive situations. Restricted pleasure or aimless (compensatory) actions, marked selfishness.

50-41 Severe symptoms, or avoidance, or antisocial behaviour/acting out or other highly inappropriate actions in response, not only to critical situations but also more ordinary challenges. Inhibited pleasure, life restrictions in several areas. Lack of sense of direction and self-realisation.

40-31 Overwhelmed by ordinary life challenges in several areas. Withdraws from most difficult situations and takes on almost no responsibilities. Severe symptoms; passive, dependent, or extremely rigid performance. Unclear communication. Severe disturbance in family life and very restricted leisure activities.

30-21 Very limited or no adaptive capacity. Cannot function adequately in almost any area. Overwhelmed by ordinary daily activities, severe disturbances in the ability to communicate.

20-11 Need assistance to solve problems of daily living. Severe self-destructive or dangerous actions.

10-1 Continuous disorganized mental functioning.
